# Muscle fat replacement and contractility in patients with skeletal muscle sodium channel disorders

**DOI:** 10.1038/s41598-023-29759-7

**Published:** 2023-02-13

**Authors:** Jonas Jalili Pedersen, Mads Godtfeldt Stemmerik, Laura Nørager Jacobsen, Sofie Vinther Skriver, Gustav Rhode Wilms, Morten Duno, John Vissing

**Affiliations:** 1 Copenhagen Neuromuscular Center, Department of Neurology, Rigshospitalet, University of Copenhagen, Inge Lehmanns Vej 7-9, 2100 Copenhagen, Denmark; 2 Clinical Genetic Laboratory, Department of Biochemical Genetics, Rigshospitalet, University of Copenhagen, Copenhagen, Denmark

**Keywords:** Neurology, Signs and symptoms

## Abstract

Skeletal muscle sodium channel disorders give rise to episodic symptoms such as myotonia and/or periodic paralysis. Chronic symptoms with permanent weakness are not considered characteristic of the phenotypes. Muscle fat replacement represents irreversible damage that inevitably will impact on muscle strength. This study investigates muscle fat replacement and contractility in patients with pathogenic *SCN4A* variants compared to healthy controls. T1-weighted and 2-point Dixon MRI of the legs were conducted to assess fat replacement. Stationary dynamometry was used to assess muscle strength. Contractility was determined by maximal muscle contraction divided by cross-sectional muscle area. The average cross-sectional intramuscular fat fraction was greater in patients compared with controls by 2.5% in the calves (95% CI 0.74–4.29%, p = 0.007) and by 2.0% in the thighs (95% CI 0.75–3.2%, p = 0.003). Muscle contractility was less in patients vs. controls by 14–27% (p < 0.05). Despite greater fat fraction and less contractility, absolute strength was not significantly less. This study quantitatively documents greater fat fraction and additionally describes difference in muscle contractility in a large cohort of patients with skeletal muscle sodium channel disorders. The clinical impact of these abnormal findings is likely limited as muscle hypertrophy in the patients served to preserve absolute muscle strength. Subgroup analysis indicated significant difference in phenotype by genotype, however these findings lack statistical significance and serve as inspiration for future researchers to probe into the geno- phenotype relationship in these disorders.

**Trial registration:** The study was registered at http://clinicaltrials.gov (identifier: NCT04808388).

## Introduction

Pathogenic variants in the *SCN4A* gene on chromosome 17q23 are associated with skeletal muscle sodium channel disorders (OMIM 603967). These autosomal dominant channelopathies include the following phenotypes in adults: paramyotonia congenita (PMC), hyperkalemic periodic paralysis (HyperPP), sodium channel myotonia (SCM) and hypokalemic periodic paralysis (HypoPP), with a combined prevalence of 1:100,000^1^. The pathogenic variants produce gain of function changes of the *SCN4A* encoded NaV-1.4 voltage-gated sodium channel and alters membrane excitability. This results in exaggerated inward Na^+^ current, which may either initiate bursts of action potentials (myotonia) or cause flaccid paralysis by depolarizing fibers to a refractory unexcitable state (periodic paralysis)^2^. Myotonia and periodic paralysis represent the typical morbidity suffered by these patients, while permanent weakness is not characteristic for this patient group. There has previously been reports indicating muscle degeneration in this patient group, but frequency, severity and pathogenesis for muscle degeneration remains undetermined^3^. MRI is established as a reliable non-invasive tool to investigate muscles in inherited neuromuscular disorders^4–9^, and new software can detect even small changes in fat fraction, which is difficult to assess qualitatively on inspection of images^10–14^. A linear relationship between muscle strength and cross-sectional area (CSA) is a hallmark of healthy muscle^15^. Muscle contractility can be defined as maximal contraction force divided by the contractile cross-sectional area (CCSA) of the muscle group producing the force. The aim of this study was to quantify muscle degeneration through quantitative MRI and relate it to muscle contractility in leg muscles of patients with *SCN4A* variants compared to healthy controls.

## Methods

### Standard protocol approvals, registrations, and patient consent

The study was approved by the Danish National Committee on health research ethics (approval number: H-18023049). The study was registered at http://clinicaltrials.gov (identifier: NCT04808388). Informed consent was obtained from all participants.

This cross-sectional observational study included 31 patients and 31 controls matched for sex, age, height, and weight. Inclusion criteria for patients were: (1) genetically verified *SCN4A* mutation and (2) age > 18. Exclusion criteria were: (1) competing muscular disorders and (2) contraindication to MRI. All data was collected and analyzed from January 2021 to January 2022.

The included patients were heterozygous for the following known pathogenic variants in *SCN4A*: p.T704M (HyperPP, n = 7), p.T1313M (PMC, n = 13), p.V1293l (PMC, n = 7), p.G1306E (SCM, n = 2) and p.V1589M (PMC, n = 2). Clinical data is summarized in Table 1.

**Table 1:** Clinical data on the 62 participants, divided into subgroups.

	G1306E (N = 2)	T1313M (N = 13)	T704M (N = 7)	V1293I (N = 7)	V1589M (N = 2)	All patients (N = 31)	Controls (N = 31)	p-value
Age								
Mean (SD)	22.0 (4.2)	49.5 (16.3)	41.6 (16.4)	34.0 (18.6)	57.0 (5.7)	42.9 (17.6)	42.4 (17.2)	0.9
Median	22.0 (19, 25)	54.0 (20, 73)	35.0 (21, 65)	28.0 (21, 75)	57.0 (53, 61)	39.0 (19, 75)	38.0 (20, 78)	
Sex								
F	1 (50.0%)	6 (46.2%)	3 (42.9%)	3 (42.9%)	1 (50.0%)	14 (45.2%)	14 (45.2%)	1
M	1 (50.0%)	7 (53.8%)	4 (57.1%)	4 (57.1%)	1 (50.0%)	17 (54.8%)	17 (54.8%)	
Height meter								
Mean (SD)	1.8 (0.1)	1.7 (0.1)	1.8 (0.1)	1.7 (0.1)	1.7 (0.1)	1.8 (0.1)	1.8 (0.1)	0.64
Median	1.8 (1.7, 1.8)	1.7 (1.6, 1.9)	1.8 (1.7, 1.9)	1.8 (1.7, 1.9)	1.7 (1.6, 1.8)	1.8 (1.6, 1.9)	1.8 (1.6, 1.9)	
Weight kilo								
Mean (SD)	70.0 (11.3)	83.2 (21.8)	77.1 (10.3)	74.6 (8.3)	91.0 (15.6)	79.5 (16.3)	74.3 (13.1)	0.16
Median	70.0 (62, 78)	83.0 (41, 125)	75.0 (66, 95)	70.0 (64, 85)	91.0 (80, 102)	79.0 (41, 125)	76.0 (53, 103)	
BMI								
Mean (SD)	22.0 (1.8)	27.5 (7.0)	23.7 (2.0)	24.5 (1.9)	30.4 (1.0)	25.8 (5.1)	23.8 (3.3)	0.7
Median	22.0 (20.7, 23.3)	28.0 (15.6, 44.3)	23.4 (21.1, 26.3)	24.5 (22.0, 27.4)	30.4 (29.7, 31.1)	25.5 (15.6, 44.3)	23.5 (18.3, 34.8)	

Age, sex, height, weight, and BMI was matched between groups (p > 0.05). SD: Standard derivation. Median inclusive minimum and maximum value. BMI: Body mass index. P-value indicate difference between all patients and control.

### Muscle MRI and mapping

The MRI protocol included axial T1-weighted and axial 2-point Dixon scans performed on a 3.0-T MRI system (Magnetom Verio Tim System: Siemens AG, Erlangen, Germany). The scans were conducted in a headfirst supine position using a spine coil, two body-matrix coils and a peripheral angio coil for the legs. For the Dixon scans, the following parameters were used: TE1 = 2.45 ms, TE2 = 3.675 ms, repetition time = 5.59 ms. Slice thickness was 3.5 mm and number of acquisitions was 300–360. Field of view was adjusted to the size of the participant ranging from 400 to 500 mm.

**Figure 1:**
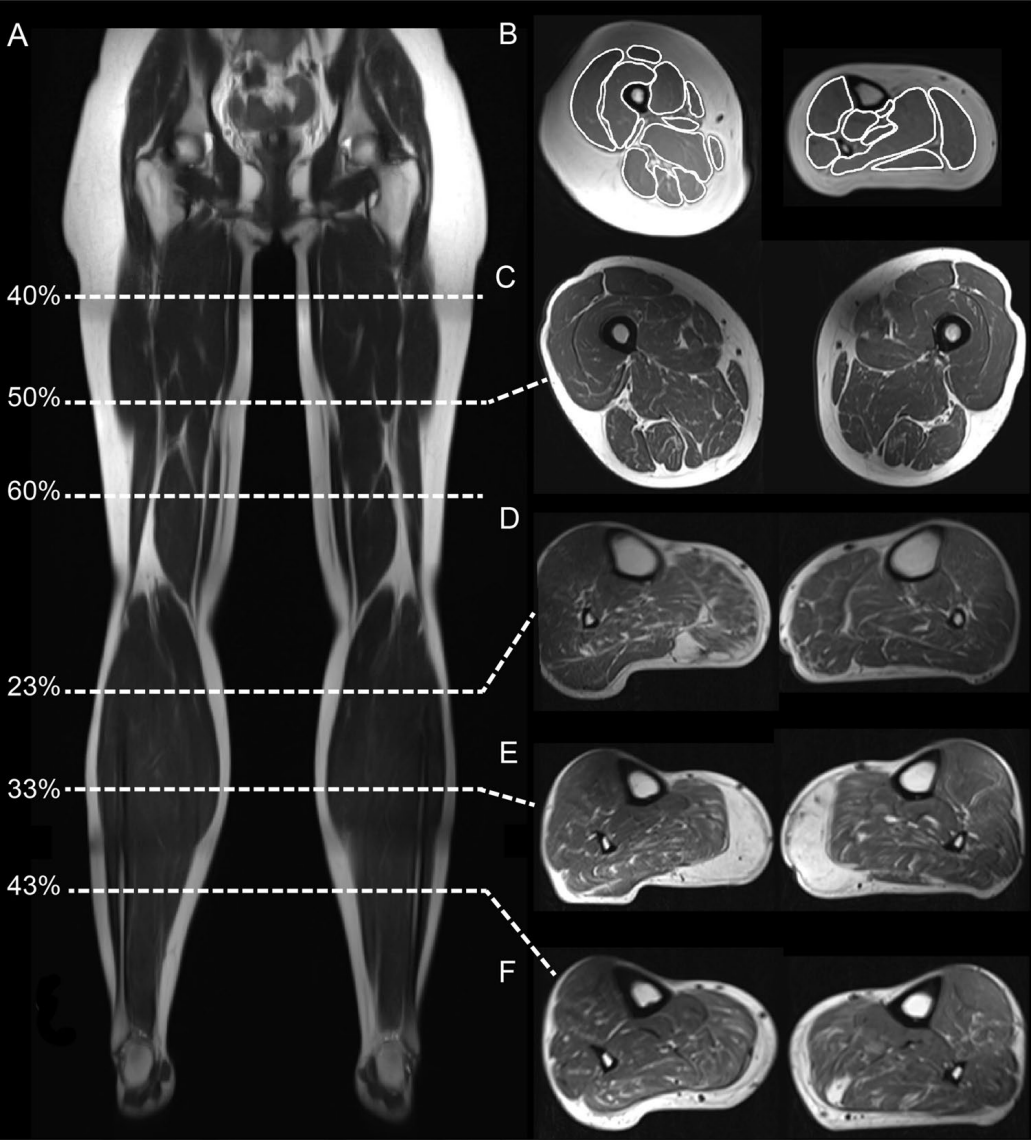
Overview of MRI. (**A**) Localizer shows the 6 different levels of segmentation on the leg. (**B**) Example of muscle mapping of the thigh (50%) and calf (33%). (**C**) T1-w MRI of a 59-year-old patient heterozygous for p.T1313M at 50% length of the thigh. (**D**–**F**) Series of T1-w MRI from the same patient at 23, 33 and 43% of the calf, demonstrating significant proximal–distal difference of involvement. In the more distal part of the muscle (at 33% length of the tibia), the gastrocnemius medialis is almost completely replaced by fat.

All images were assessed using an open-source medical imaging viewer for MacOS (Horos™ V. 4.0), in which muscles were mapped manually with a mouse tool. Both legs were imaged. Since there was no side difference in term of fat fraction on visual inspection, we only analyzed one leg. The leg chosen for segmentation was the same as the one used for stationary dynamometry. To investigate heterogeneous involvement of fat replacement, and to make sure we found the largest contractile cross-sectional area (CSA) for contractility measures, each muscle was mapped at three different levels. This means that 3 axial slices of the thigh corresponding to 40, 50 and 60% of femur length (measured from caput femoris), and 3 axial slices of the calf corresponding to 23, 33 and 43% of tibia length (measured from tibial plateau) were chosen for muscle segmentation (Fig. 1A). To avoid inclusion of confounding inter-fascicular fatty tissue from, muscles were mapped individually on each section, instead of in groups (Fig. 1B). Due to natural anatomical muscle origin and insertion, musculus biceps femoris brevis was not detectable proximally on the 40% femur section, and adductor longus was not detectable distally on the 60% femur section (Fig. 1C) Therefore, those muscles did not contribute with data points at above mentioned levels. In the thigh, adductor longus and adductor brevis (collectively “*adductors*”) were mapped together because their facias were indistinguishable on most of the images. In the calf, tibialis anterior, extensor digitorum longus and extensor hallucis longus (collectively “*anterior calf group*”), as well as peroneus brevis and longus (collectively “*peroneus*”) were mapped together because their facias were also indistinguishable on most of the images. In total, each participant contributed with 372 axial sections, amounting to roughly 3472 individual muscles regions mapped from the 62 participants. To avoid observational bias, all images were anonymized in the mapping phase, so that the analyst was blinded to whether scans represented controls or patients.

### Muscle strength evaluation

Maximal isometric muscle strength measured by peak torque (PT) in Newton-meter (Nm) was assessed using a stationary dynamometer (Biodex Medical System 4 PRO, NY, USA). The Biodex stationary dynamometry system is a reliable tool for evaluating muscle strength in patient with neuromuscular disorders, and has been used in several other similar studies^10,11,15^. For all individuals, their preferred leg was chosen for dynamometry, unless they had a chronic injury in that leg. Flexion and extension of the knee and ankle joints were isolated by stabilizing shoulder, abdomen, and legs with straps and lever arms attached to the Biodex system. There was no movement during the test, as the Biodex system registers stationary force. The test included 3 attempts of 5 s maximal contraction in both direction of each of the two investigated joints (knee and ankle). A threshold of 15% coefficient of variance was predetermined to exclude unreliable measurements, however all participants performed better than this coefficient for all muscle groups. One patient was excluded from muscle strength assessment due to pain from a herniated spinal disk, and 2 other patients had to be excluded due to local software replacement at time of evaluation.

### Muscle-fat replacement calculation

The term muscle-fat replacement is a commonly used descriptor of fat in muscles where muscle tissue was present earlier, without indicating the mechanism by which this replacement happened. The 2-point Dixon protocol provides 4 images represented by: (1) *fat-phase*, (2) *water-phase*, (3) *in-phase* (fat + water) and (4) *out-phase* (fat–water). Horos™ is a software that provides information about signal intensity (SI) and cross-sectional area (mm^2^) for each mapped region. To calculate fat fraction (FF), we used the signal intensity of a *fat-phase* image divided by the SI of the corresponding *in-phase* image: $FF=\frac{SI\left(fat only\right)}{SI(fat+water)}*100$. The fat fraction analysis was separated into two categories: sectional analysis and individual muscle analysis. To determine a general difference in fat replacement the first analysis looked at the average cross-sectional fat fraction on each of the 6 sections (Fig. 1A). To determine whether some muscles were prone to more severe fat replacement, the second analysis looked at the fat fraction of individual muscle on each section.

### Muscle contractility

Contractility is referred to as peak torque divided by contractile cross-sectional area. The term normalized strength could have been used interchangeable, but in this paper, we refer to it as contractility. To evaluate the fat-fat free lean muscle area of the cross-sectional area (CSA) (by excluding the intramuscular fatty tissue), contractile cross-sectional area (CCSA) was defined as: $CCSA=CSA*\frac{1-FF}{100}$. In this way, only the functional muscle tissue of each participant was included in the contractile analysis. Contractility was then calculated as peak torque divided with lean muscle area (CCSA): $contractility=\frac{PT (Nm)}{CCSA (m{m}^{2})}$.

Both the thigh and calf were divided into two functional muscle groups corresponding to their function: (1) flexor group thigh (biceps femoris, semimembranosus, semitendinosus, gracilis and sartorius) (2) extensor group thigh (rectus femoris, vastus intermedius, vastus lateralis, vastus medialis) (3) flexor group calf (gastrocnemius lateralis, gastrocnemius medialis, soleus, flexor hallucis longus, flexor digitorum longus and tibialis posterior) (4) extensor group calf (tibialis anterior, extensor digitorum longus and extensor hallucis longus). Previous studies have indicated that the cross-sectional area near 50% length of the muscle is a good compromise to represent the full volume in leg muscles^16,17^. To investigate if this was true for individuals in this group, we investigated the cross-section with the largest CCSA for each muscle group. We considered the section with the largest muscle area to be most appropriate for calculating contractility.

### Questionnaires and myotonic tests

For secondary outcomes, we wished to evaluate the subjective and clinical impact of disease. All patients were tapered out of their myotonic medicine 5 days prior to evaluation. Two questionnaires and several functional tests were conducted. The Myotonia Behavior Scale (MBS) evaluates the subjective nuisance from myotonia on a scale from 0 to 5^18^. MBS score of 0 corresponds to no myotonia, while a score of 5 corresponds to debilitating myotonia in daily life. Individualized Neuromuscular Quality of Life (INQoL) questionnaire evaluates disease impact regarding both personal and social life^19^. An INQol score of 0 reflects no disease impact on quality of life, while a score of 100 reflects severe impact. A series of functional tests were used to evaluate time to relaxation after 5-s contractions of the eyelid, hand, and jaw muscles. These squeeze tests were repeated 5 times for jaw and eyelid, and 10 times for the hands to take into account that paradoxical myotonia sometimes present after repetitive movements in the PMC patients^20^. To evaluate myotonia in the legs, we used the Timed Up and Go (TUG) and Sitting and Rising Test (SRT). In TUG, the participants were instructed to stand up, walk 6 m and sit back again. In the SRT, participant stood up to full extension and sat back again for as many times as possible in 10 s.

### Statistical analysis

The open-source statistical application Rstudio (V 4.1.2) was used for statistical analysis. For data with repeated measures due to several segmentation on each patient (FF and CSA), we used a linear mixed-effects model in the data analysis. All data were tested for normal distribution, and log-transformation was applied when data were not normally distributed. For non-repeated measures, we used the independent *T-test* for parametric data, and the *Mann–Whitney-U-test* for non-parametric data. *Pearson r* was used to test correlations between two numeric variables. A p-value of ≤ 0.05 was considered significant. All p-values were Bonferroni-corrected corresponding to the number of tests made on the same data sample.

### Ethical approval statement

All procedures followed were in accordance with the ethical standards of the responsible committee on human experimentation (institutional and national) and with the Helsinki Declaration of 1975, as revised in 2000^5^ (approval number: H-18023049).

### Informed consent

Informed consent was obtained from all patients for being included in the study.

## Results

### Cross-sectional analysis

**Figure 2:**
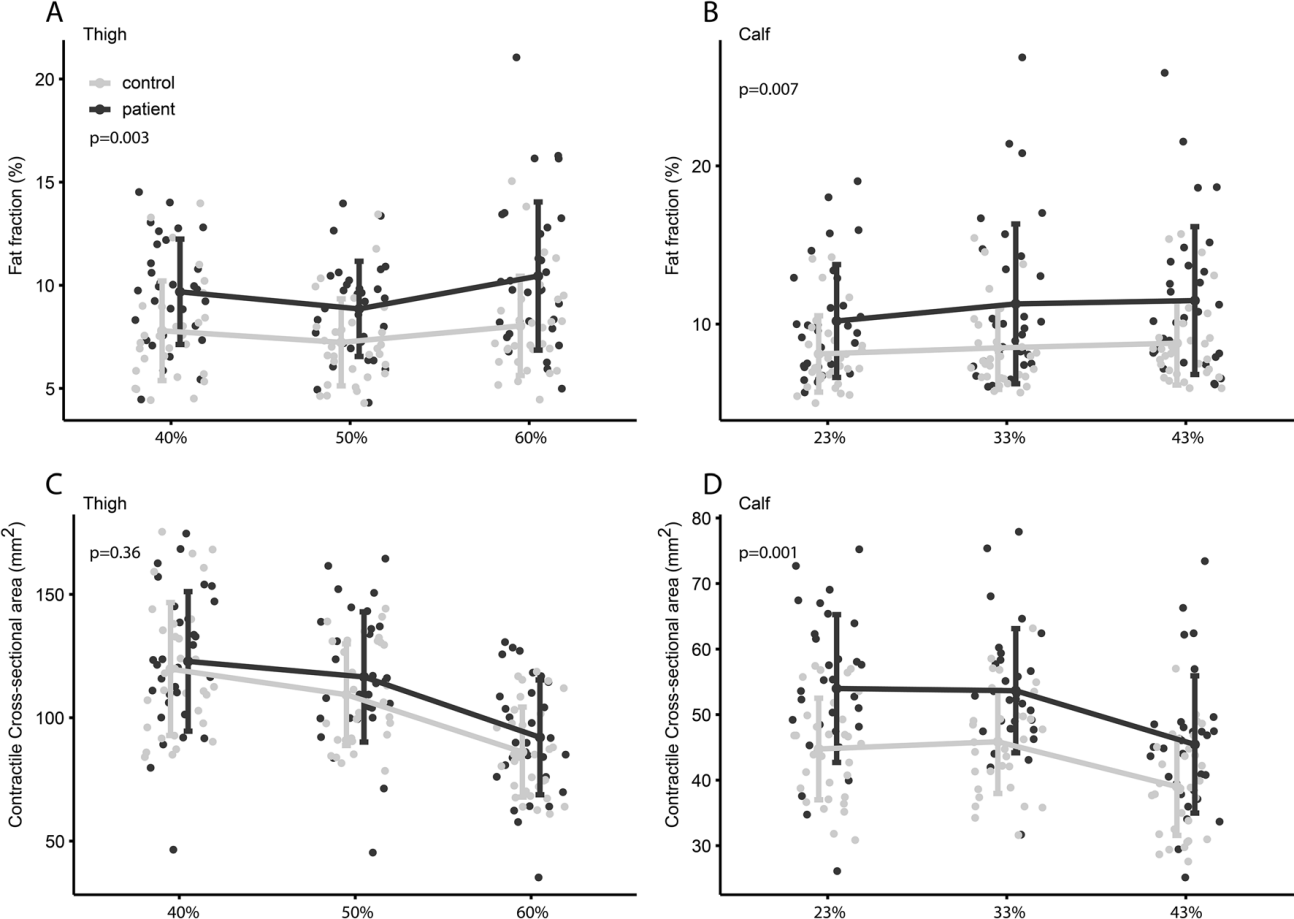
Fat fraction (FF) and contractile cross-sectional area (CCSA) on the different segments. (**A**,**B**) Average fat fraction of calf and thigh. Y-axis is fat fraction in percentage. The difference in FF between the proximal segments and distal segments is significant (p < 0.05). (**C**,**D**) Average CCSA on cross sections. Y-axis CCSA in mm^2^. CCSA was significantly higher for patients in the calf, but only nominally so in the thigh, indicating hypertrophy of lower leg muscles.

Fat fraction (FF) was mildly greater in patients vs. controls by 2.5% in the calves (95% CI 0.74–4.29%, p = 0.007) and by 2.0% in the thighs (95% CI 0.75–3.2%, p = 0.003) (Fig. 2). In both patients and controls, there were a distal–proximal gradient for (FF) in both thighs and calves, which was significant using a mixed effect model comparing the proximal with the distal segment (p < 0.005). The patient group had significantly greater CSA by 23% in the calves (95% CI 5.7–16.1 mm^2^, p < 0.0001) and nominally so by 9% in the thighs (95% CI − 2.3 to 21.3 mm^2^, p = 0.12). The fat-free muscle area, CCSA, was also significantly higher by 18% in the calves (95% CI 3.6–12.1 mm^2^, p = 0.001) and nominally so by 5% in the thighs (95 CI − 6.2 to 17.1 mm^2^, p = 0.366).

### Individual muscle analysis

**Figure 3:**
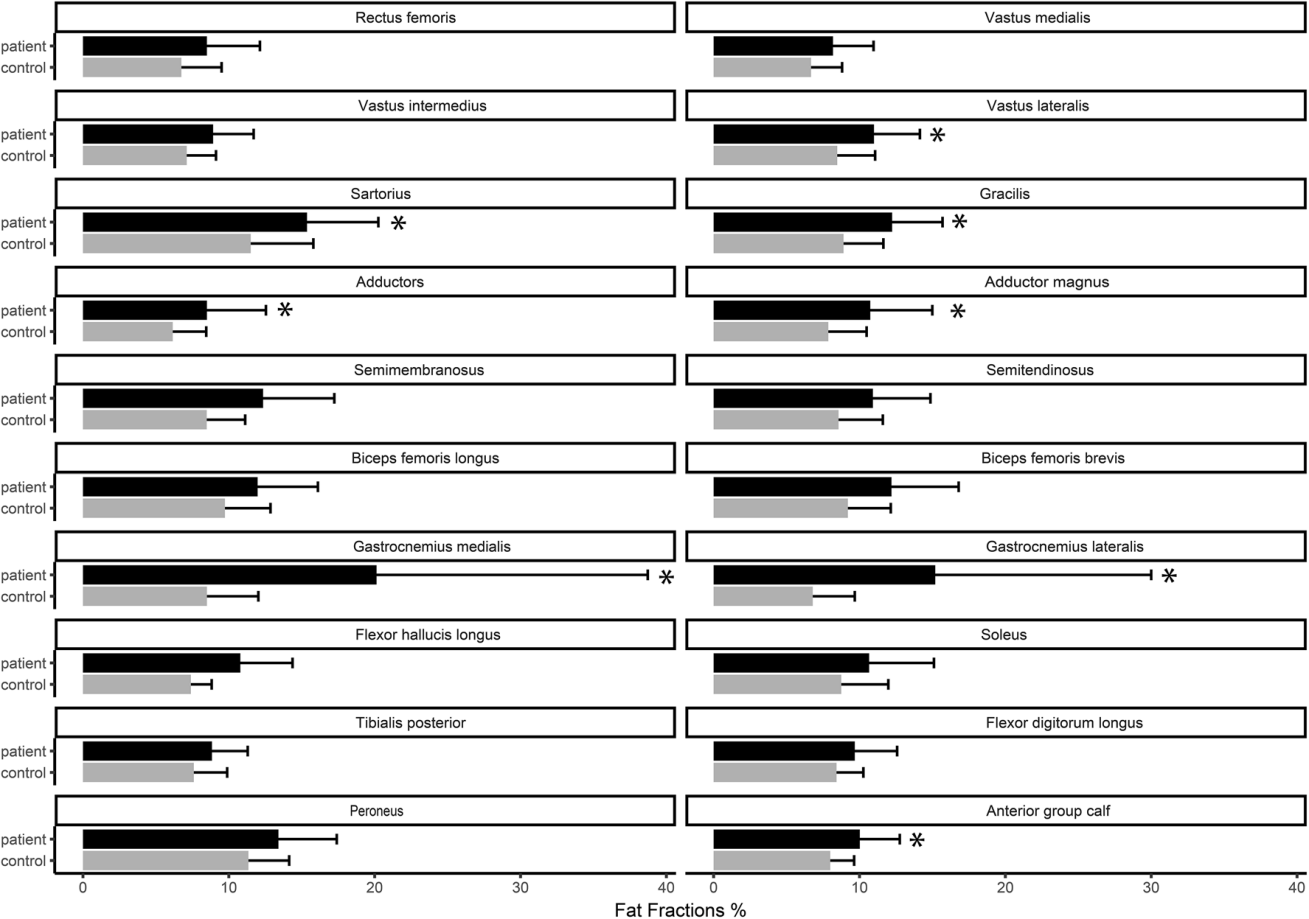
Fat fraction of single muscles. Fat fraction of individual muscles in patients with SCN4A mutations and healthy controls. Error bars represent standard deviation. Muscles with significant difference between patients and controls are marked with an asterisk (p < 0.05).

A significant difference in FF was found in 3/8 muscles of the calf (gastrocnemius lateralis, gastrocnemius medialis, and *anterior group*) along with 5/12 muscles of the thigh (vastus lateralis, gracilis, sartorius, *adductors*, and adductor magnus) (Fig. 3). The largest difference in FF was found in the gastrocnemius medialis (11%, 95 CI 4.4–17.6%, p = 0.01) and gastrocnemius lateralis (7%, 95% CI 2.3–11.7%, p = 0.04). The difference in FF between the proximal and distal sections of the gastrocnemii muscles were significant; gastrocnemius medialis 13.0% (95 CI 7.1–19.0, p < 0.05) and gastrocnemius lateralis 14.1% (95% CI 6.3–22.0, p < 0.05), emphasizing a heterogenous involvement of these muscles (visualized in Fig. 1D–F).

### Muscle contractility

**Figure 4:**
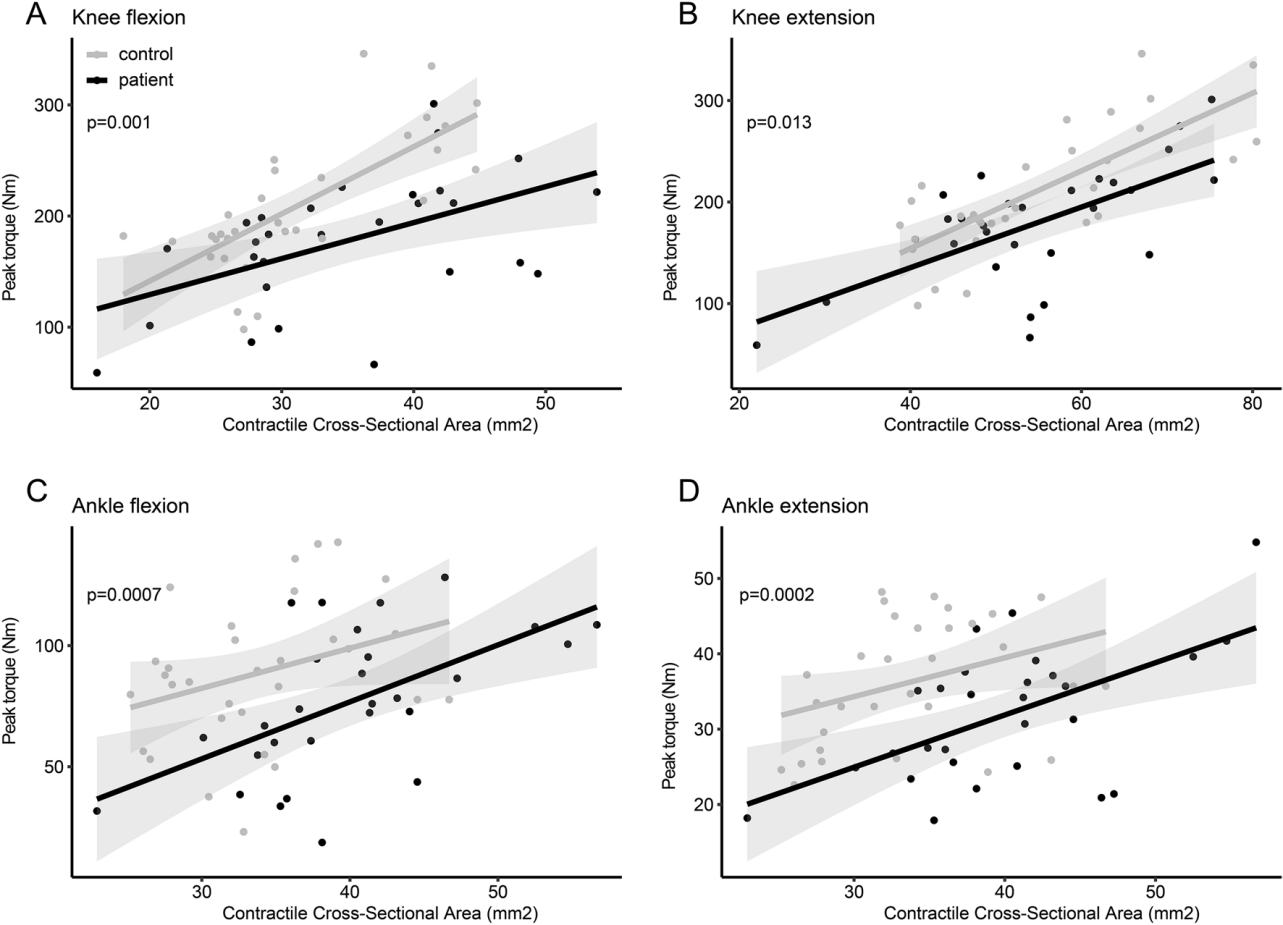
Contractility of the four studied muscle groups. Peak torque (Newton-meter)) divided by contractile cross-sectional area (mm^2^) reflects contractility. The patients have significantly less contractility in all four studied muscle groups (p < 0.005).

**Figure 5:**
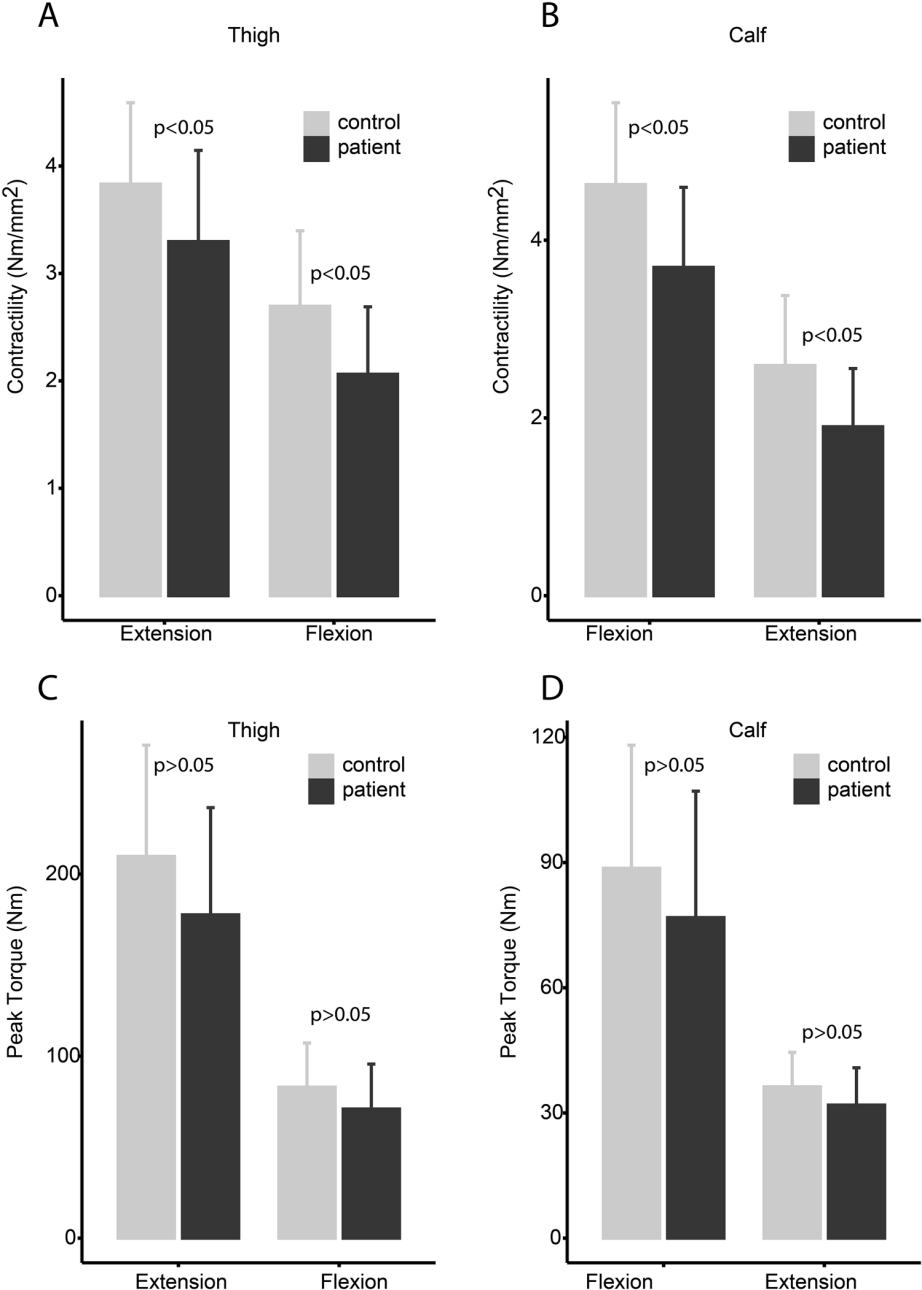
Bar plots of contractility and absolute strength between patients and controls. There was a significant difference in contractility for all muscle groups, while absolute strength was only nominally less. Our hypothesis is that increased contractile cross-sectional area compensate for reduced contractility, thus resulting in normalized absolute strength.

The region of each muscle group with the largest CCSA was found at: thigh extensors 40% of femur length, thigh flexors at 60% of femur length. Bot calf plantar and dorsal flexors were largest at 33% of tibia length. Contractility was significantly less in all 4 studied muscle groups in patients vs. controls: knee flexion − 23% (95% CI 0.3–1.0 Nm/mm^2^, p = 0.0005), knee extension − 14% (0.1–1.0 Nm/mm^2^, p = 0.01), ankle plantar flexion − 27% (95% CI 0.3–1.1 Nm/mm^2^, p = 0.006), ankle dorsal flexion − 20% (95% CI 0.5–1.4 Nm/mm^2^, p = 0.002). There was a positive correlation between CCSA and muscle strength for both patients and controls, but contractility was shifted downwards in the patient group, indicating perturbed muscle contractility (Fig. 4). The patients’ maximal muscle strength (Newton-meter), however was only insignificantly less by 13–15% in all 4 studies muscle groups (p > 0.05) (Fig. 5). There was only little or no correlation between contractility and FF as only one muscle group showed significant correlation for the patients: flexor group thigh, R = − 0.58, p = 0.0012.

### Explorative analysis

**Figure 6:**
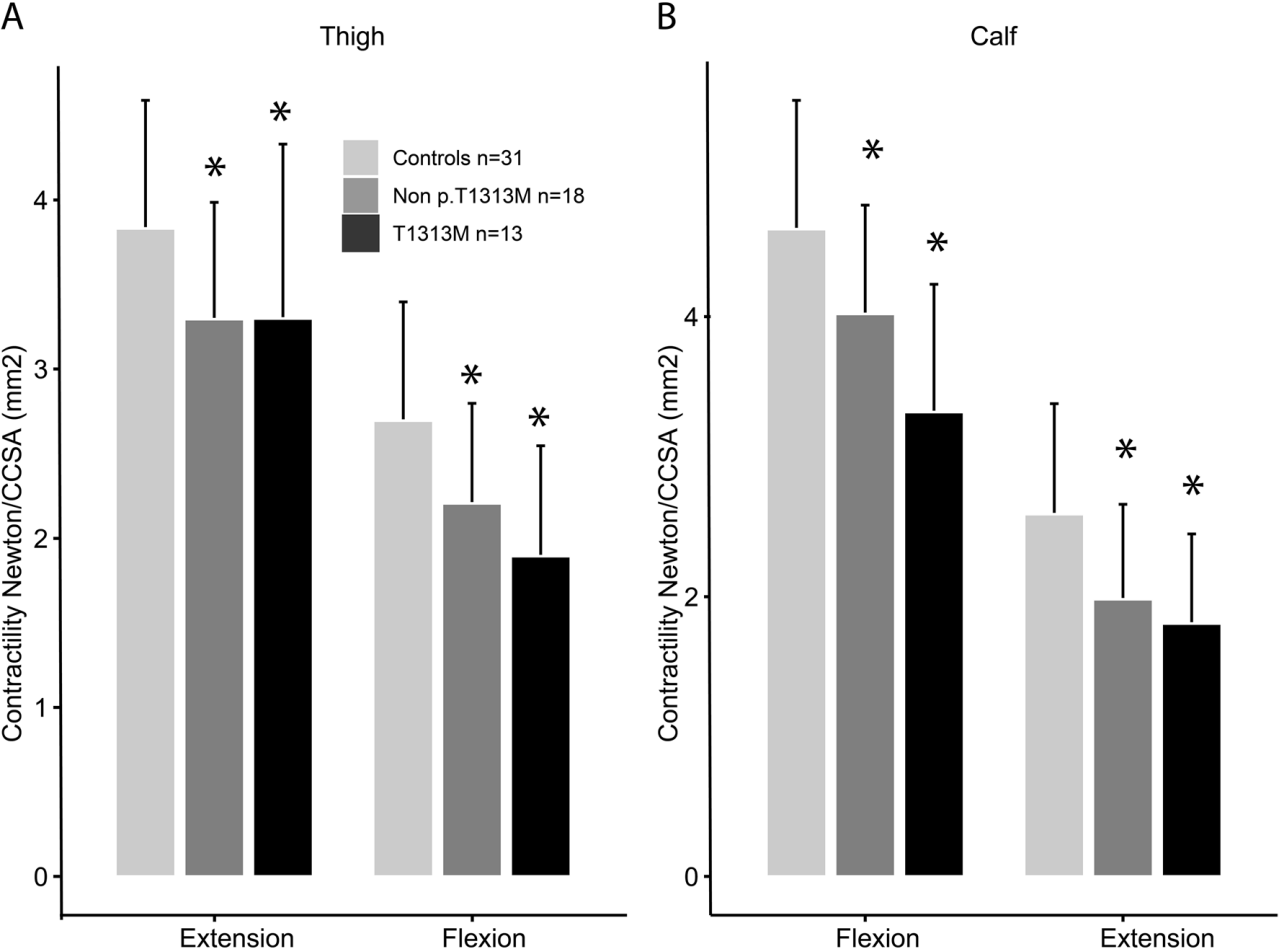
Bar plots of contractility in subgroups consisting of p.T1313M variants and non-T1313M variant group controls. Error bars are standard deviation. Both p.T1313M and the non-T1313M variants had significantly less contractility in all studied muscle groups (asterisk; p < 0.05).

Evaluating the T1-weighted images qualitatively, we found that 8/31 patients and 0/31 controls presented with gastrocnemii muscles that were visually completely washed out by fat (Mercuri Score 4, end-stage appearance) as demonstrated in Fig. 1E. Of these patients, 7/8 were heterozygous for the p.T1313M mutation (n = 13). Due to the natural development of the data, we conducted a subgroup analysis of the non-p.T1313M genotypes pooled together (n = 18) and found that the non-p.T1313M patients had comparable muscle FF compared with controls. When conducting the same subgroup analysis on contractility, however, both subgroups (p.T1313M and non-p.T1313M) showed significantly less contractility compared to controls in all 4 functional muscle groups (Fig. 6). These findings led us to do further explorative analysis on FF in genotype groups. Of the three subgroups with at least 7 patients (T1313M, V1293, T704M), the only genotype group that showed significantly greater muscle FF on sectional analysis when compared to controls was the p.T1313M group. These latter results, however, should be interpreted with caution and are reported without statistical significance due to very small sample sizes in the subgroups.

### Clinical evaluation

Self-reported myotonia did not correlate with muscle FF in the calf (r = 0.24, p = 0.19) or thigh (r = 0.31, p = 0.093). Additionally, age did not correlate with myotonia, quality of life or contractility, but correlated positively with FF in both thigh (r = 0.72, p < 0.05) and calf (r = 0.65, p < 0.05). Data from the clinical evaluation is summarized in Table 2. The INQoL questionnaire showed that quality of life was impacted in patient on average (18/100) and was worst in the p.T1313M group, indicating a higher subjective impact of disease. Patients with the p.T1313M genotype also had more myotonia both clinically and self-reported.

**Table 2:** Data on clinical test and questionnaires.

Test	V1293I (N = 7)	T704M (N = 7)	T1313M (N = 13)	V1589M (N = 2)	G1306E (N = 2)	All (N = 31)	Controls (N = 31)
Phenotype	Paramyotonia congenita	Periodic paralysis	Paramyotonia congenita	Paramyotonia congenita	Sodium channel myotonia		
Eyelid relaxation time, s (SD)	1.4 (0.76)	0.7 (0.14)	12.3 (11.10)	9.4 (3.1)	61.5 (20.8)	10.2 (16.8)	0.8 (0.15)
Hand relaxation time, s (SD)	1.2 (0.37)	0.7 (0.13)	46.8 (25.5)	10.3 (8.4)	4.8 (1.6)	21.0 (27)	0.7 (0.15)
Jaw relaxation time, s (SD)	1.9 (1.63)	0.7 (0.10)	4.8 (4.7)	4.7 (9.8)	31.2 (5.4)	4.8 (8.0)	0.8 (0.17)
TUG outside reference	0/7	0/7	0/13	1/2	0/2	1/31	0/31
SRT outside reference	2/7	2/7	6/13	2/2	1/2	13/31	0/31
MBS average (0–5)	1.3	2.4	4.0	2	2.5	3.0	–
INQol average	13	8	28	13.5	17.2	18	–

Relaxation times are mean. SD: Standard derivation. TUG: Timed Up and Go. SRT: Sitting and Rising Test. MBS: Myotonia Behavior Scale (score 0–5, as 5 represent debilitating impact of myotonia on daily life). INQoL: Individualized Neuromuscular Quality of Life (score 0–100, as 100 represent very low quality of life). Note that results from the two small genotype groups (p.V1589M n = 2, p.G1306E n = 2) represent very small sample sizes. Both objectively and subjectively, patient heterozygous for p.T1313M suffer from significant myotonia. This patient group also scores worse on quality of life (28).

## Discussion

The present study demonstrates greater muscle degeneration and less muscle contractility in patients with skeletal muscle sodium channel disorders compared to healthy controls. The study further shows that there was a heterogeneity in muscle involvement, with the most notable muscle wasting found in the gastrocnemii muscles of the calf. This study also evaluated several segments along the thigh and calf, which revealed a heterogenous involvement of individual muscle with progressive worsening of fat replacement distally comparted to proximal slices. Interestingly, despite the difference in contractility, the patients had close to normal maximal strength compared to controls. This is likely due to compensation from muscle hypertrophy in the patient group, documented by greater contractile cross-sectional area. The patients show a preserved relationship between increasing muscle area and strength, as observed in healthy individuals. Muscle hypertrophy is a clinically well described phenomenon in patients with non-dystrophic myotonias^21,22^. Thus, even though fat fraction was greater, and contractility was less, these quantitative results do not suggest the presence of permanent weakness besides the well-known characteristics of myotonia and/or periodic paralysis in this patient group.

We did not find a significant correlation between muscle fat fraction and contractility. Similar quantitative MRI study have investigated fat replacement and contractility in patients with spinobulbar muscular atrophy^11^ and Becker muscular dystrophy^15^. The studies described a clear correlation between greater fat replacement and less contractility. A correlation that seems logical in the presence of much higher levels and more diffuse pattern of muscle-fat replacement, something that was not the case with these *SCN4A* individuals. The present findings indicate that the less contractility may be explained by other mechanism than a mildly increased muscle degeneration. Cannon et al. studied the possible link between different mutant ion channels and muscle excitability of patients with skeletal muscle sodium channel disorder^2^. The sodium leak present in these disorders results in either slow inactivation of the sodium channel and eventual myotonic bursts of discharge (myotonia), or incomplete inactivation of the channel resulting in persistent inward current and a stable depolarized refractory unexcitable state (periodic paralysis). We propose that the mechanic effects of impaired synchronic muscle excitability may be the cause of less muscle contractility demonstrated in this patient group. Anatomical differences in muscle structure, for example pennation of the muscle, could influence on muscle contractility, however we have no evidence to suggest that gross anatomical structure of the muscles differs between patients and controls. The pathogenesis of muscle degeneration in *SCN4a* is still unknown^3^ but few studies have indicated that there might be a molecular involved in muscle-fat replacement. A study from Miller et al. on patients with skeletal muscle sodium channel disorders found that 67% of patients (n = 82) with hyperkalemic periodic paralysis phenotype presented with a vacuolar myopathy on muscle biopsy^23^. A recent study on another non-dystrophic myotonia group, the skeletal muscle calcium channel disorders, hypokalemic periodic paralysis (HypoPP), have presented biopsy results showing large vacuoles consisting of accumulated glycogen and giant autophagosomes likely resulting from perturbations of the autophagy flux^24^. Whether pathological vacuoles in present patients also stem from perturbations of the autophagy flux is uncertain, but it would be highly interesting to investigate this in future studies and may lead to greater understanding of the pathogenesis of muscle degeneration.

Other methods have previously been used to address muscular changes in patients with *SCN4A* mutations. Trip et al. used ultrasound to investigate a pooled group of *SCN4A* individuals and showed greater mean echo intensity indicating muscle degeneration^25^. Morrow et al. used T1-weighted MRI and STIR (Short Tau Inversion Recovery) in a case series to demonstrate inflammation and edema also indicating muscle degeneration^26^. A more similar study investigated 7 patient with hyperkalemic paralysis using Dixon MRI^27^, and proposed “evidence for chronic progressive myopathy with selective muscle involvement”. These previous studies were, however, limited by small sample sizes and did not test contractility.

This study was powered to do an analysis primarily on the whole cohort of SCN4A variants. Due to the natural development of the data however, indicating subgroup difference by genotype, we felt obliged to touch on genotype analysis. However, these results lack statistical power, and we know little about the genotype variability in these disorders. This may explain the discrepancy between previous and present findings in patients with p.T704M variant^27^. The 7 p.T704M patients included in present study did not present with significant difference in muscle degeneration compared to controls. On the contrary, this study indicate a much larger impact in patient with the p.T1313M mutation, as more than half of these variants (7/13) had end stage appearance with washed out gastrocnemii muscles on T1-w imaging (Mercuri Score^7^). One patient outside the p.T1313M group presented with the same pattern (male 75 y, genotype p.V1293I). Subgroup analysis showed that the p.T1313M group seemed to be driving most of the difference in fat fraction in this specific group. Interestingly, both the p.T1313M and non-p.T1313M groups had less muscle contractility, indicating that all patients have less muscle contractility regardless of muscle fat fraction. These results further support the previous statement, that less muscle contractility could be explained by other mechanism such as impaired synchronic muscle contraction. A figure showing subgroup analysis by genotype can be found in supplementary materials, however this data was excluded from the main manuscript due to lack of statistical power.

Quality of life was more severely influenced in patients with the p.T1313M mutation. This can most likely be explained by the fact that this a phenotype with severe daily myotonia, impacting on everyday activity. Another reasonable way to subdivide the patients would be by phenotype, thus investigating if patients with myotonia have more or less fat fraction compared to patients with periodic paralysis. It is tempting to propose that myotonia may relate to muscle degeneration because of daily involuntary muscle strain. However, we did not find a general significant correlation between reported myotonia and greater muscle fat fraction. Another study on HypoPP patients have demonstrated muscle fat replacement on aging, even though these patients doesn’t have myotonia at all^28^. As stated previously, the prior study on the 7 patients with hyperkalemic periodic paralysis also suggested muscle degeneration, even though that phenotype doesn’t suffer prom myotonia either^27^. Considering previous and present findings it seems unlikely that muscle degeneration can be explained by stress from myotonia.

Some key features of channelopathies are that they are characterized by paroxysmal attacks and that symptoms tend to decline over time as seen in other ion channel disorders such as epilepsy and migraine^29^. In the present study, we found that myotonia, contractility and quality of life neither worsened nor declined with age, while fat fraction had a very significant positive correlation with age. The average age of the 8 patients with completely degenerated gastrocnemii muscles was 60 years, and the mean age of the 7 p.T704M not showing significant degeneration was 42 years. Thus we cannot exclude that the difference in muscle fat replacement between p.T704M and p.T1313M may merely be explained by an age difference. We propose that these disorders are only affected by symptomatic paroxysmal symptoms such as myotonia or periodic paralysis, but also hold an element of underlying chronic affection with irreversible transformation of muscle to fat. While the clinical symptoms don’t seem to be age dependent, progressive myopathy probably is.

The present study quantitatively documents greater fat replacement of muscles and impaired muscle contractility in patients with skeletal muscle sodium channel disorders. However, due to muscle hypertrophy, these changes do not confer significant clinical weakness. Thus, these findings likely added little quality of life disability to the main symptoms of myotonia and/or periodic paralysis in these patients. Additionally, this study present remarkable apparent affection of a particular genotype, as this is an inspiration for other researchers to probe into the geno- phenotype relationship in these disorders. In future studies, it would be interesting to follow these abnormal findings in muscles of patients with sodium channelopathies longitudinally.

## Supplementary Information

Supplementary Information 1.

Supplementary Figure 1.

## Data Availability

Data supporting this study is presented in the result section. Patient specific data can be made available in anonymized form upon reasonable request, taking in regard GDPR regulations. Data can be made available by contact with the corresponding author: Jonas Jalili Pedersen, Jalilijonas@gmail.com.
