# Supplementary material for: Muscle fat replacement and contractility in patients with skeletal muscle sodium channel disorders
Source: Sci Rep. 2023 Feb 13;13:2538. doi: 10.1038/s41598-023-29759-7 (PMC9925746; doi:10.1038/s41598-023-29759-7)
Supplement: Supplementary file 1 — Supplementary Information 1. [file 41598_2023_29759_MOESM1_ESM.docx]

Text for supplementary graph on genotype subgroups:

“Subgroup analysis indicated difference in phenotype by genotype. Y-axis is average fat fraction in percentage, while x-axis represents the 6 subgroups including controls. Figure A represents fat fractions in the thigh, Figure B represents fat fractions in the calf. The boxplot holds the median and interquartile range, while the vertical line represents maximum and minimum values. For full transparency the figure holds all 3 datapoints from all individuals (3 slices along thigh and calf). The figure serves to give the reader an insight on the significant difference in phenotype by genotype. These data were not used in the statistical analysis of the main manuscripts, due to lack of power for statistical significance in the small subgroups.”
