# Supplementary figures and images for: Muscle fat replacement and contractility in patients with skeletal muscle sodium channel disorders
Source: Sci Rep. 2023 Feb 13;13:2538. doi: 10.1038/s41598-023-29759-7 (PMC9925746; doi:10.1038/s41598-023-29759-7)

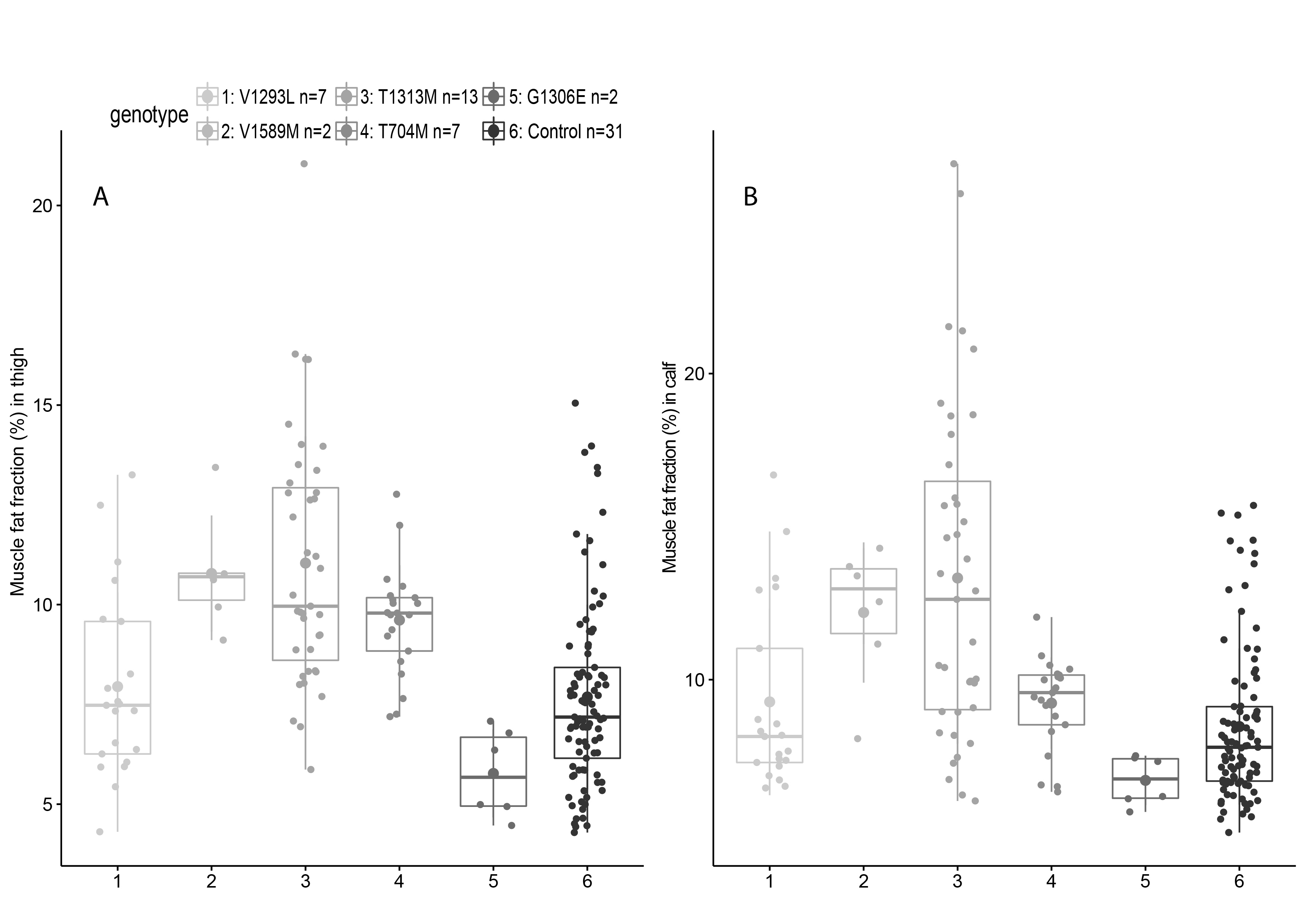

Supplement: Supplementary file 2 — Supplementary Figure 1. [file 41598_2023_29759_MOESM2_ESM.jpg]
